# Whole-Genome Sequence of Paenibacillus polymyxa Strain SRT9.1, a Promising Plant Growth-Promoting Bacterium

**DOI:** 10.1128/mra.01097-21

**Published:** 2022-01-20

**Authors:** Tshifhiwa P. Mamphogoro, Casper N. Kamutando, Martin M. Maboko, Olubukola O. Babalola, Olayinka A. Aiyegoro

**Affiliations:** a Gastro-Intestinal Microbiology and Biotechnology Unit, Agricultural Research Council—Animal Production, Irene, Pretoria, South Africa; b Department of Plant Production Sciences and Technologies, University of Zimbabwe, Mount Pleasant, Harare, Zimbabwe; c Department of Crop Sciences, Tshwane University of Technology, Pretoria, South Africa; d Food Security and Safety Niche Area, Faculty of Natural and Agricultural Sciences, North-West University, Mmabatho, South Africa; e Research Unit for Environmental Sciences and Management, North-West University, Potchefstroom, South Africa; Montana State University

## Abstract

Paenibacillus polymyxa SRT9.1 is an epiphytic bacterium capable of inhibiting plant-pathogenic bacteria. The strain has potential for development as a biocontrol agent for use in agriculture. We report the whole-genome sequence of Paenibacillus polymyxa SRT9.1, consisting of 6,754,470 bp and 7,878 coding sequences, with an average G+C content of 45%.

## ANNOUNCEMENT

*Paenibacillus polymyxa*, a Gram-positive bacterium, has long been used as a phytopathogen biological control agent and plant growth and development booster. The plant growth-promoting and biocontrol activity of Paenibacillus polymyxa against plant pathogens is attributed to antimicrobial compound production, nitrogen fixation, the secretion of hydrolytic enzymes, and siderophore production (1–4).

*P*. *polymyxa* SRT9.1 was isolated from the surface of fungicide-treated red sweet peppers sourced from the Agricultural Research Council—Vegetable and Ornamental Plants Institute (ARC-VOP), Roodeplaat, Pretoria, South Africa (25°59′S, 28°35′E; altitude, 1,200 m above sea level). Fresh peppers, grown in open soil conditions, were collected in sterile, sealed plastic bags (5). Bacterial biofilms on the surfaces of the pepper fruits were recovered using sterile cotton swabs soaked in a solution containing 0.15 M NaCl and 0.1% vol/vol Tween 20 (6). The swabs were vortexed in sterile Eppendorf tubes containing saline solution (0.85% [vol/vol] NaCl). The supernatant was serially diluted, plated onto Trypticase soy agar (TSA), and incubated for 48 h at 30°C under aerobic conditions. Distinct colonies were streaked onto TSA. *P. polymyxa* SRT9.1 was taxonomically identified as in references 7 and 8. The strain tested positive for *in vitro* plant growth-promoting abilities such as siderophore production and phosphate solubilization (3). The pure strain was subcultured in Trypticase soy broth (TSB), incubated at 30°C for 48 h, and stored in 50% (vol/vol) glycerol at −80°C. Revived *P. polymyxa* SRT9.1 was then subcultured on TSA and the biomass used for DNA extraction.

Genomic DNA of the SRT9.1 strain was extracted using the Quick-DNA fungal/bacterial miniprep kit (Zymo Research, Irvine, CA; catalog number D6005) and sequenced on the Illumina NextSeq platform at Inqaba Biotechnical Industries (Pty) Ltd. (Pretoria, South Africa). The NEBNext Ultra II FS DNA library prep kit (New England Biolabs, Ipswich, MA) was used for DNA library preparation. The libraries were then sequenced on the Illumina NextSeq 550 platform, and a total of 2,224,054 reads generated from each sample (2 × 150-bp paired-end reads) were visualized using the KBase platform (9). The quality of the reads was evaluated using FastQC v0.11.5 (10), while low-quality reads and sequence adaptors were removed using Trimmomatic v0.36 (11). The genome assembly was performed using SPAdes v3.13.0 (12) with default parameters, yielding 3,172 contigs with a coverage of 100×. The assembly yielded a genome seqeuence of 233 Mbp, 6,754,470 bp long, with a G+C content of 45.0%. The contigs had *N*_50_ and *L*_50_ values of 219,142 bp and 9, respectively. The contigs were annotated using the Rapid Annotations Subsystems Technology v1.073 toolkit (13) and the publicly available NCBI Prokaryotic Genome Annotation Pipeline (14), which identified 7,878 protein-coding genes, 63 RNA genes, 12 rRNA genes, 46 tRNA genes, and 136 pseudogenes. Additionally, secondary metabolites for antagonism of Fusarium graminearum (e.g., polymyxin and fusaricidin) (15) were identified using antiSMASH v5.0.0 (16). A whole-genome-based phylogenic tree for SRT9.1 was constructed using the Type (Strain) Genome Server (17), and SRT9.1 showed close similarity with Paenibacillus polymyxa ATCC 842 (GenBank accession number AFOX01000032.1), with a digital DNA-DNA hybridization (dDDH) value of 99.9% to the type strain.

### Data availability.

This whole-genome shotgun project has been deposited at DDBJ/ENA/GenBank under the accession number JAJFEV000000000. The version described in this paper is the first version. The SRA accession number is SRR16351787, the BioProject accession number is PRJNA771517, and the BioSample accession number is SAMN22314579.
